# Evolutionary Adaptation of the Essential tRNA Methyltransferase TrmD to the Signaling Molecule 3′,5′-cAMP in Bacteria*[Fn FN2]

**DOI:** 10.1074/jbc.M116.758896

**Published:** 2016-11-23

**Authors:** Yong Zhang, Rym Agrebi, Lauren E. Bellows, Jean-François Collet, Volkhard Kaever, Angelika Gründling

**Affiliations:** From the ‡ Section of Microbiology and MRC Centre for Molecular Bacteriology and Infection, Imperial College London, London SW7 2AZ, United Kingdom,; § WELBIO, Avenue Hippocrate 75, 1200 Brussels, Belgium,; ¶ de Duve Institute, Université Catholique de Louvain, Avenue Hippocrate 75, 1200 Brussels, Belgium, and; ‖ Research Core Unit Metabolomics, Hannover Medical School, Carl-Neuberg-Str. 1, 30625 Hannover, Germany

**Keywords:** cyclic AMP (cAMP), gram-positive bacteria, protein evolution, signaling, *Staphylococcus aureus* (*S. aureus*)

## Abstract

The nucleotide signaling molecule 3′,5′-cyclic adenosine monophosphate (3′,5′-cAMP) plays important physiological roles, ranging from carbon catabolite repression in bacteria to mediating the action of hormones in higher eukaryotes, including human. However, it remains unclear whether 3′,5′-cAMP is universally present in the Firmicutes group of bacteria. We hypothesized that searching for proteins that bind 3′,5′-cAMP might provide new insight into this question. Accordingly, we performed a genome-wide screen and identified the essential *Staphylococcus aureus* tRNA m^1^G37 methyltransferase enzyme TrmD, which is conserved in all three domains of life as a tight 3′,5′-cAMP-binding protein. TrmD enzymes are known to use *S*-adenosyl-l-methionine (AdoMet) as substrate; we have shown that 3′,5′-cAMP binds competitively with AdoMet to the *S. aureus* TrmD protein, indicating an overlapping binding site. However, the physiological relevance of this discovery remained unclear, as we were unable to identify a functional adenylate cyclase in *S. aureus* and only detected 2′,3′-cAMP but not 3′,5′-cAMP in cellular extracts. Interestingly, TrmD proteins from *Escherichia coli* and *Mycobacterium tuberculosis*, organisms known to synthesize 3′,5′-cAMP, did not bind this signaling nucleotide. Comparative bioinformatics, mutagenesis, and biochemical analyses revealed that the highly conserved Tyr-86 residue in *E. coli* TrmD is essential to discriminate between 3′,5′-cAMP and the native substrate AdoMet. Combined with a phylogenetic analysis, these results suggest that amino acids in the substrate binding pocket of TrmD underwent an adaptive evolution to accommodate the emergence of adenylate cyclases and thus the signaling molecule 3′,5′-cAMP. Altogether this further indicates that *S. aureus* does not produce 3′,5′-cAMP, which would otherwise competitively inhibit an essential enzyme.

## Introduction

3′,5′-Cyclic adenosine monophosphate (3′,5′-cAMP) is a second messenger molecule found in all three domains of life (1). It is involved in the regulation of a variety of physiological processes ranging from carbon catabolite repression (CCR)^3^ in bacteria to mediating the action of hormones in eukaryotes (1). CCR exists in most bacteria and describes the phenomenon that certain carbon sources (usually glucose) are preferentially catabolized over other secondary carbon sources. This is achieved through complex positive and negative regulatory transcription networks (2). CCR is well studied in *Escherichia coli*, where 3′,5′-cAMP and the catabolite receptor protein (CRP) together form an active transcriptional factor that activates the expression of genes coding for proteins involved in catabolizing secondary carbon sources when glucose is exhausted (2). 3′,5′-cAMP is synthesized from ATP by adenylate cyclases (ACs), a large family of enzymes with divergent sequence, domain, and structural features (1). So far, six classes of ACs have been reported, with class III enzymes found in all domains of life and classes I, II, and IV only present in bacteria (1). The ACs identified in *Prevotella ruminicola* and *Rhizobium etli* are distinct from the existing families and were proposed to form Class V and VI enzymes, respectively (3, 4). Class I ACs are exemplified by the *E. coli* CyaA enzyme; class II ACs are bacterial toxins most often secreted into eukaryotic host cells where they perturb host cell functions (5). Lastly, class IV ACs are a unique group of proteins only found in bacteria but forming part of a larger protein family called CYTH domain proteins (6, 7). CYTH proteins are an ancient protein family that exists in all three domains of life and are named after the type IV AC CyaB from *Aeromonas hydrophila* and the human thiamine triphosphatase. It has been proposed that these enzymes were originally inorganic tripolyphosphatases and subsequently evolved to contain other enzymatic activities such as adenylate cyclase, mRNA triphosphatase, and thiamine triphosphatase activity (6, 7). CYTH proteins contain a characteristic and highly conserved E*X*E*X*K amino acid motif at their N terminus and have a conserved fold with eight β-sheets forming a tunnel-like structure (6). Various other conserved charged amino acids with their side chains projecting into the tunnel have been identified, and these are involved in coordinating the different polyphosphate substrates or are involved in enzyme catalysis (6, 8–10).

Although ACs enzymes are in general widely distributed among bacteria, there is conflicting evidence if 3′,5′-cAMP is produced and plays a physiological role in the Firmicutes group of bacteria. Although a bioinformatics analysis performed by Galperin *et al.* (11) on 555 complete bacterial and archaeal proteomes indicated that adenylate cyclase enzymes are absent in the majority of Firmicutes bacteria, including *Staphylococcus aureus*, a protein corresponding to SACOL1008 of *S. aureus* strain COL is nevertheless often annotated as adenylate cyclase. However, the predicted cyclase activity of this protein has never been tested. It has also been reported that 3′,5′-cAMP is present in *Bacillus subtilis* when grown under oxygen limitation conditions, and its level was shown to decrease in the presence of nitrate (12, 13). However, in these studies the molecule suggested to be 3′,5′-cAMP was identified only through chromatographic methods and its actual chemical structure was never confirmed by other methods, such as mass spectrometry, which is now routinely used. In *S. aureus*, ArcR (SACOL2653 in strain COL), a member of the CRP/FNR family of bacterial transcriptional regulators, plays a role in mediating catabolite repression by inducing the arginine deiminase operon genes *arcABDC* under anaerobic conditions (14). Furthermore, 3′,5′-cAMP was shown in *in vitro* assays to enhance the ability of ArcR to bind to the promoter region of the *lctE* gene, coding for an l-lactate dehydrogenase (14). However, it was never tested if 3′,5′-cAMP is actually present in *S. aureus* and has a similar effect on ArcR *in vivo*. In this study we wanted to shed further light on whether or not 3′, 5′-cAMP is produced and plays a physiological function in *S. aureus*. Using an *S. aureus* ORFeome protein expression library, we screened for 3′,5′-cAMP-binding proteins and identified TrmD as a tight 3′,5′-cAMP-binding protein. However, we were unable to detect 3′,5′-cAMP under various growth conditions nor a functional adenylate cyclase in *S. aureus*. TrmD is a highly conserved tRNA methyltransferase and present in all three domains of life (15). It converts Gly-37 into m^1^G37 by transferring the methyl group from S-adenosylmethionine (AdoMet) to a subset of tRNA species (15, 16). We further found that TrmD proteins from *E. coli* and *Mycobacterium tuberculosis* do not bind 3′,5′-cAMP. Subsequent bioinformatics and extensive biochemical analyses suggested that 3′,5′-cAMP competes with AdoMet for binding, presumably inhibiting the essential function of TrmD in *S. aureus*. Together with a phylogeny analysis, our data suggest that 3′,5′-cAMP is absent in *Staphylococcus*. Finally, our work also highlights that the emergence of 3′,5′-cAMP as a signaling molecule in bacteria required essential evolutionary adaptations of AdoMet-binding proteins such as TrmD.

## Results

### 

#### 

##### Identification of the S. aureus TrmD Protein as a 3′,5′-cAMP-binding Protein

**FIGURE 1.**
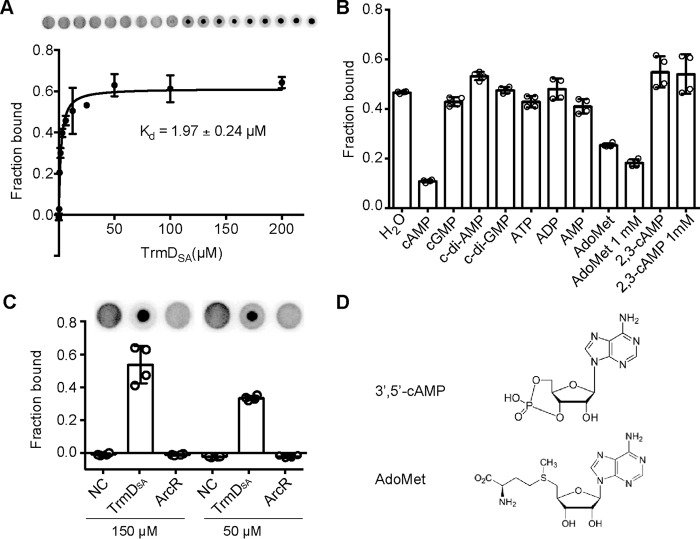
**TrmD from *S. aureus* binds 3′,5′-cAMP with high affinity.** *A*, binding curve and *K_d_* determination between 3′,5′-cAMP and TrmD_SA_. Radiolabeled 3′,5′-cAMP and purified His-TrmD_SA_ protein ranging from 1.5 nm to 200 μm were used in DRaCALAs, the average fraction-bound values and S.D. of four replicates were plotted, and the curve-fitted and *K_d_* values were determined as previously described (17). Representative DRaCALA spots are shown on *top of the graph. B*, nucleotide binding competition assays. A 100 μm concentration of purified His-TrmD_SA_ protein was incubated with radiolabeled 3′,5′-cAMP (∼2 nm) and 0.4 mm concentrations of the indicated cold competitor nucleotide or 0.4 mm or 1 mm AdoMet and 2′,3′-cAMP. The average fraction-bound values and S.D. from four spots were determined and plotted. *C*, the *S. aureus* CRP-like transcription factor ArcR does not bind 3′,5′-cAMP. Radiolabeled 3′,5′-cAMP was incubated as the negative control (*NC*) in the absence of protein or purified TrmD_SA_ or ArcR proteins (from two different purifications) at a concentration of 150 or 50 μm as indicated. Average fraction-bound values and S.D. from four experiments are plotted. *D*, chemical structures of 3′,5′-cAMP and AdoMet.

There is conflicting evidence whether or not 3′,5′-cAMP exists in the Firmicutes group of bacteria. We reasoned that if 3′,5′-cAMP is indeed present and functionally relevant in *S. aureus*, specific 3′,5′-cAMP-binding protein(s) must exist. To investigate this, we made use of a *S. aureus* ORFeome protein expression library and the differential radial capillary action of ligand assay (DRaCALA), a simple and fast method for the detection of small molecule-protein interactions (17–19). The DRaCALA method is based on the principle that free radiolabeled small ligand will diffuse outward once spotted on a nitrocellulose membrane but will stay as a tight spot when bound to a protein (17). In previous studies this assay and the ORFeome protein expression library was successfully used to identify c-di-AMP and ppGpp-binding proteins in *S. aureus* (18–20). The ORFeome protein expression library is a collection of 2337 *E. coli* strains allowing for the overproduction of 86% of the annotated *S. aureus* strain COL proteins as His-MBP-fusion proteins (18–20). To apply this assay to the identification of potential 3′,5′-cAMP-binding proteins, the expression of the *S. aureus* proteins was induced, and *E. coli* cell lysates were prepared. Next, radiolabeled [^32^P]cAMP was synthesized using a C-terminal truncated form of the *E. coli* adenylate cyclase enzyme CyaA. As assessed by thin layer chromatography (TLC), 97% of the input [α-^32^P]ATP was converted to [α-^32^P]cAMP (data not shown). The genome wide DRaCALA screen was subsequently performed as previously described (18–20). Two technical replicates were performed, and the lysate from one strain expressing the *S. aureus* COL protein SACOL1256 (plate 13 well F05) gave a positive result for 3′,5′-cAMP binding (data not shown). SACOL1256 (or SAUSA300_1133 in the USA300 strain FPR3757) codes for the tRNA methyltransferase TrmD, termed from here on out as TrmD_SA_. To further investigate if TrmD_SA_ can bind 3′,5′-cAMP with a physiological relevant affinity, SAUSA300_1133 from the USA300 strain LAC* was cloned into vector pET28b, and the protein was expressed and purified as N-terminally His-tagged fusion protein. DRaCALAs were carried out with [^32^P]cAMP and serially diluted TrmD_SA_ protein ranging from 200 μm to 1.5 nm and a *K_d_* of 1.97 ± 0.24 μm was determined (Fig. 1*A*). This binding affinity is in a similar range as the reported *K_d_* of 2 μm for the interaction between the *E. coli* transcription factor CRP and 3′,5′-cAMP (21). To test if the binding is specific to 3′,5′-cAMP, an excess of the cold competitor nucleotides 3′,5′-cAMP, 3′,5′-cGMP, c-di-GMP, and c-di-AMP was added to the binding reaction. This analysis revealed that only cold 3′,5′-cAMP but none of the other nucleotides tested could compete for binding with the radiolabeled [^32^P]cAMP (Fig. 1*B*). Two forms of cAMP have been detected in cells, 3′,5′-cAMP, the classic signaling nucleotide, and 2′,3′-cAMP, suggested to be a nucleotide intermediate formed during the RNA degradation process (22, 23). As revealed by competitive binding assays, only the classic signaling molecule 3′,5′-cAMP but not 2′,3′-cAMP could prevent the binding of radiolabeled 3′,5′-cAMP to TrmD_SA_ (Fig. 1*B*). Taken together, these data show that the *S. aureus* TrmD protein is able to bind the 3′,5′-cAMP signaling nucleotide with high affinity. However, it is also of note that no interaction between 3′,5′-cAMP and the *S. aureus* ArcR protein, a transcription factor with homology to the *E. coli* CRP protein, could be detected (Fig. 1*C*).

##### 3′,5′-cAMP Competitively Binds at the AdoMet Substrate Binding Site of the S. aureus TrmD Protein

TrmD is a highly conserved and essential enzyme and present in nearly all bacteria. TrmD is responsible for methylating the Gly-37 residue at the N^1^ position in a subset of tRNAs using AdoMet as methyl donor (15). Because 3′,5′-cAMP is chemically similar to AdoMet and both contain an adenine moiety and a ribose ring (Fig. 1*D*), this raised the possibility that 3′,5′-cAMP binds at the AdoMet substrate binding site of TrmD_SA_. Indeed, as revealed by a competitive binding assay, AdoMet could inhibit in a dose-dependent manner the binding of radiolabeled 3′,5′-cAMP to TrmD_SA_ (Fig. 1*B*). Next, isothermal titration calorimetry (ITC) experiments were performed, and a *K_d_* of 121.4 μm was determined for AdoMet binding to TrmD_SA_. Of note, the TrmD_SA_ and AdoMet interaction was determined by ITC and not DRaCALA, as the latter method can only be used for high affinity binding interactions (low μm *K_d_* or below) and using a radiolabeled ligand. Taken together, these data indicate that 3′,5′-cAMP binds at the same site as the natural substrate AdoMet, consistent with a competitive binding mechanism.

##### 2′,3′-cAMP, but Not 3′,5′-cAMP, Can Be Detected in S. aureus Extracts

**FIGURE 2.**
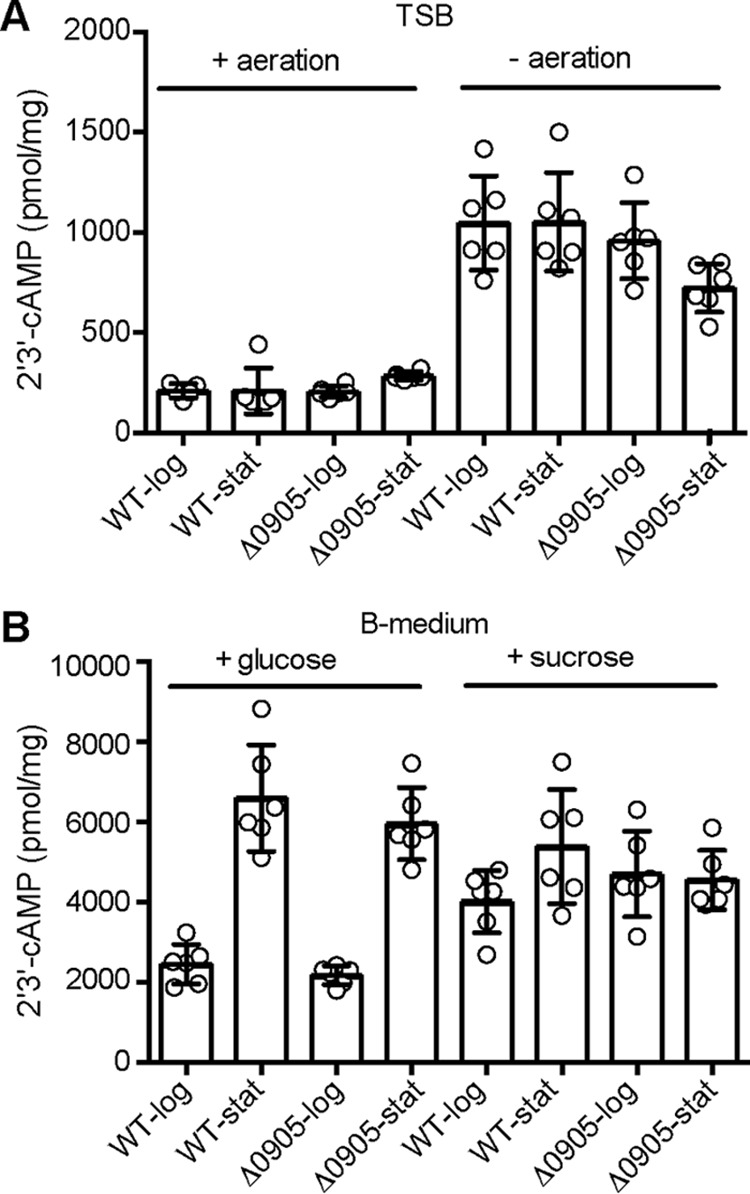
**2′,3′-cAMP, but not 3′,5′-cAMP, is present in *S. aureus*.** Semiquantitative measurements of 2′,3′-cAMP in extracts prepared from wild-type *S. aureus* strain JE2 and the isogeneic SAUSA300_0905 mutant strain (Δ0905). Bacteria were grown in TSB medium with/without agitation (*A*) or in B-medium supplemented with 25 mm of glucose or sucrose (*B*). As indicated in the graph, extracts were either prepared from logarithmic (log) phase cultures or stationary (stat) phase cultures.

Previous work indicated that cAMP is produced in *B. subtilis* when grown without aeration under oxygen limitation conditions (12, 13). However, it should be noted that cAMP production was only assessed using chromatographic methods, and its chemical structure was never confirmed by NMR- or mass spectrometry-based methods (12, 13). Given the fact that we uncovered a 3′,5′-cAMP-binding protein in *S. aureus*, we next set out to determine if and when 3′,5′-cAMP is produced in *S. aureus* using a sensitive mass spectrometry-based method (24). Cytosolic extracts were prepared from the wild-type *S. aureus* strain JE2 and strain NE1299, containing a transposon insertion in SAUSA300_0905, coding for an uncharacterized protein often annotated as adenylate cyclase. The strains were grown in tryptic soy broth (TSB) medium under aerobic or micro-aerobic conditions as well as in B-medium supplemented with either glucose or sucrose as the only carbon source to reflect carbon catabolite repression in *S. aureus* (25). Extracts were prepared from both exponential and stationary phase cultures, and nucleotides were detected by LC-MS/MS as described in Bähre and Kaever (24). Using this sensitive method, 3′,5′-cAMP concentrations can be detected up to a lower limit of 0.412 pmol per sample and also discriminated from 2′,3′-cAMP. Large amounts of 2′,3′-cAMP were detected in all samples (Fig. 2). Normalization based on total protein concentrations revealed higher 2′,3′-cAMP levels in extracts prepared from strains grown in TSB medium under micro-aerobic than under aerobic conditions (Fig. 2). The 2′,3′-cAMP levels were even higher when bacteria were grown in B-medium (Fig. 2). Of note, more 2′,3′-cAMP was also present in extracts prepared from stationary than exponential phase cells, when the bacteria were grown in B-medium supplemented with glucose. Although high levels of 2′,3′-cAMP could be detected in all samples, 3′,5′-cAMP was not detected in any of the extracts, suggesting that *S. aureus* does not produce 3′,5′-cAMP at least under the conditions tested.

##### SAUSA300_0905 and Its Homologs Are Distinct from Type IV AC Enzymes

**FIGURE 3.**
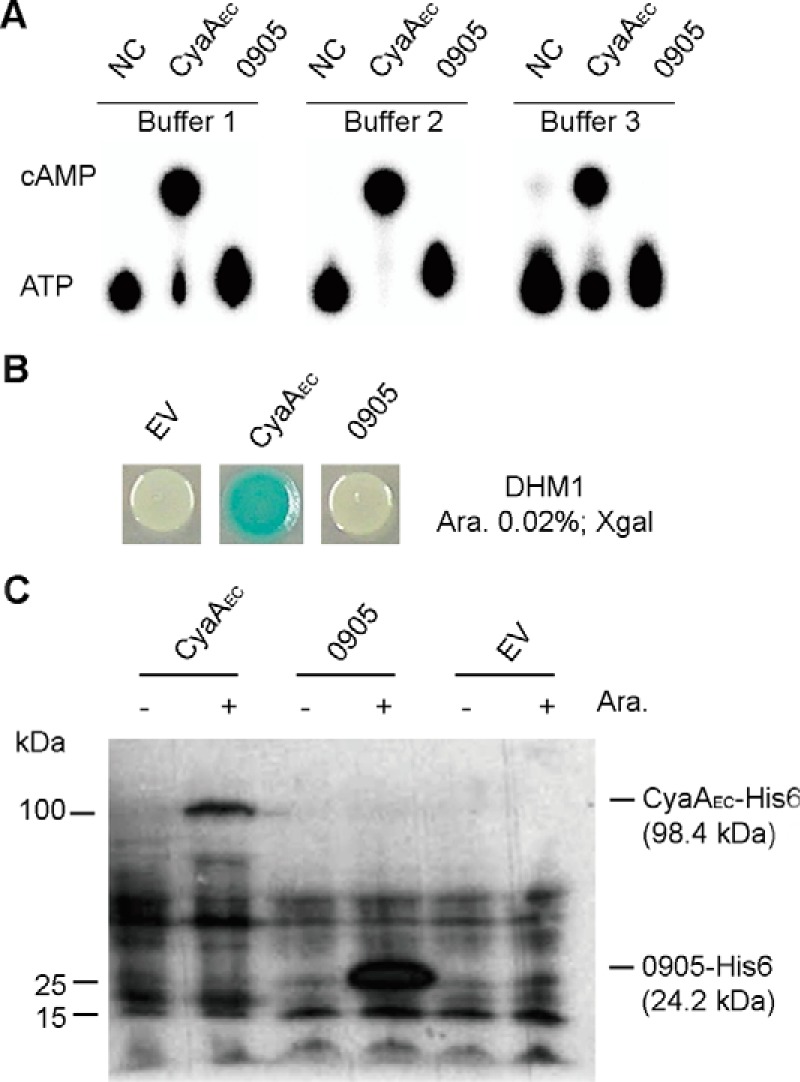
**SAUSA300_0905 is likely not a genuine adenylate cyclase.** *A*, *in vitro* adenylate cyclase activity assay. Radiolabeled ATP was incubated as the negative control (*NC*) in the absence of protein and as the positive control with purified *E. coli* CyaA(2–446) protein (CyaA_EC_) or with purified histidine-tagged SAUSA300_0905 protein (0905). The reactions were set up in three different buffer systems as specified under “Experimental Procedures” and incubated at 37 °C for 1 h. Aliquots were separated by TLC, and radiolabeled compounds visualized using phosphorimaging. A representative result of three experiments is shown. *B*, *in vivo* adenylate cyclase activity. The empty vector pBAD33 (*EV*) and plasmids pBAD-*cyaA_EC_*(2–446)-His_6_ (CyaA_EC_) or pBAD-SAUSA300_0905-His_6_ (0905) were introduced into the *cyaA* mutant *E. coli* strain DHM1. The transformants were spotted onto LB plate supplemented with 50 μg/ml X-Gal and 0.02% arabinose. *C*, detection of CyaA_EC_-His_6_ and SAUSA300_0905-His_6_ by Western blot. *E. coli* DHM1 containing plasmid pBAD33, pBAD-*cyaA_EC_*-His_6_, or pBAD-SAUSA300_0905-His_6_ was propagated in LB medium without or with 0.02% arabinose. Whole cell lysates were prepared as described under “Experimental Procedures,” proteins were separated on a 12% SDS-PAGE gel, and His-tagged proteins were detected using a His-tag-specific antibody.

An initial bioinformatics analysis indicated that the predicted *S. aureus* adenylate cyclase SAUSA300_0905 (USA300 FPR3757 nomenclature) is most closely related to type IV AC enzymes. It is possible that the SAUSA300_0905 protein was not expressed or active under the growth conditions tested, and hence, we were unable to detect 3′,5′-cAMP in the *S. aureus* extracts. To test if SAUSA300_0905 is able to synthesize 3′,5′-cAMP *in vitro*, the protein was expressed and purified from *E. coli* as the N-terminal His-tag fusion protein. The *E. coli* CyaA_2–446_ protein was purified and used as positive control. Radiolabeled [α-^32^P]ATP was used as the substrate, and reactions were set up in three different buffers, as previously reported for *in vitro* enzyme assays with the *E. coli* CyaA (26) or the *Yersinia pestis* type IV AC CyaB (8). The enzyme reactions were incubated for 1 h or overnight at 37 °C, and the reaction products were subsequently analyzed by TLC. Within 1 h, the *E. coli* CyaA_2–446_ enzyme converted >91 and 61% of the ATP to cAMP in the Mg^2+^- and Mn^2+^-containing buffers, respectively; however, none of the [α-^32^P]ATP was converted by SAUSA300_0905 (Fig. 3*A*). Overnight reactions essentially yielded similar results (data not shown). Genes coding for active AC enzymes have previously been identified through their ability to complement an *E. coli cyaA* mutant strain using simple plate assays, as 3′,5′-cAMP-producing *E. coli* strains appear red or blue on MacConkey- or X-Gal-containing plates, respectively (3, 4, 27). To test if SAUSA300_0905 is able to produce 3′,5′-cAMP when expressed in *E. coli*, SAUSA300_0905 and as a positive control the *E. coli cyaA*_2–446_ gene were cloned with an N-terminal His tag in vector pBAD33 and expressed under the control of the arabinose-inducible promoter. The resulting plasmids, pBAD33-SAUSA300_0905-His_6_ and pBAD33-*cyaA*_2–446_-His_6_, and the empty vector pBAD33 as negative control were introduced into the *cyaA* mutant *E. coli* strain DHM1 and plated on LB agar plates supplemented with 10 μg/ml chloramphenicol, 0.02% w/v arabinose, and 50 μg/ml X-Gal. Introduction of plasmid pBAD33-CyaA_2–446_-His_6_ in strain DHM1 yielded solid blue colonies, whereas the empty vector and pBAD33-SAUSA300_0905-containing cells gave white colonies (Fig. 3*B*). Western blot analysis confirmed that both proteins, CyaA_2–446_-His_6_ and SAUSA300_0905-His_6_, were expressed (Fig. 3*C*). These data indicate that SAUSA300_0905 may not be an active adenylate cyclase.

**FIGURE 4.**
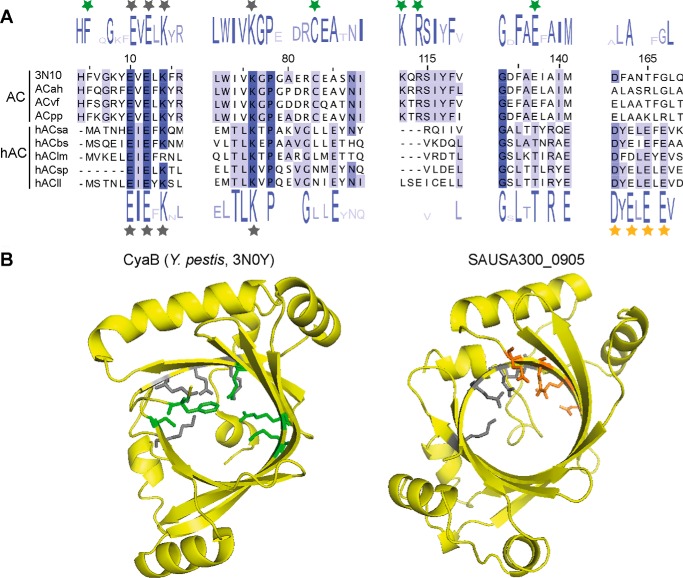
**SAUSA300_0905 is distinct from genuine type IV adenylate cyclases.** *A*, partial sequence alignment and Logo motifs based on amino acid identity for *Y. pestis* CyaB and *S. aureus* SAUSA300_0905 homologs. Homologs of CyaB and SAUSA300_0905 were identified by a BLASTP search, and multiple sequence alignments were performed within and across the two groups as outlined under “Experimental Procedures.” Key sections of the sequence logo motifs for CyaB homologs (and likely genuine adenylate cyclases (*AC*)) and SAUSA300_0905 homologs (hypothetical adenylate cyclases (*hAC*)) are shown *above* or *below the alignment*, respectively. The sequences of a few representative proteins are shown, which include: 3N10, the CyaB adenylate cyclase class IV from *Y. pestis*; ACah, WP_048207795.1, adenylate cyclase from *A. hydrophila*; ACvf, WP_044367144.1, adenylate cyclase from *Vibrio fluvialis*; ACpp, CAG22648.1, putative adenylate cyclase CyaB from *Photobacterium profundum* SS9; hACsa, SAUSA300_0905 from *S. aureus*; hACbs, KJJ42798.1, hypothetical protein UM89_04625 from *B. subtilis*; hAClm, CCO63569.1, uncharacterized protein YjbK from *Listeria monocytogenes* LL195; hACsp, CIQ15488.1, adenylate cyclase from *Streptococcus pneumoniae*; hACll, WP_010905301.1, adenylate cyclase from *Lactococcus lactis. Gray stars* indicate residues conserved in both groups of proteins; *green stars* indicate residues specific for homologs of genuine adenylate cyclases; *orange stars* indicate amino acids conserved in SAUSA300_0905 and its homologs. *B*, structural model of the *S. aureus* protein SAUSA300_0905. *Left panel*: structure of the adenylate cyclase enzyme CyaB from *Y. pestis* (PDB code 3N0Y) with key amino acid residues involved in ligand binding shown in a *stick representation. Right panel*: structural model of the *S. aureus* protein SAUSA300_0905 predicted with Phyre2 and modeled on bh2851, a putative adenylate cyclase from *B. halodurans* (PDB code 2GFG). 94% of residues modeled at >90% confidence. *Gray sticks* indicate residues conserved in both groups of proteins; *green* and *orange sticks* indicate highly conserved residues specific for CyaB and SAUSA300_0905 homologs, respectively.

To investigate this further, we revisited the annotation of SAUSA300_0905 by performing a detailed bioinformatics analysis. SAUSA300_0905 was used in a BLASTP search, the result of which indicated that it belongs to the CYTH superfamily of protein similar to *bona fide* type IV adenylate cyclases. Next, homologs of SAUSA300_0905 were retrieved from the NCBI non-redundant (nr) protein sequence database, and this yielded 998 sequences with a minimum of 30% sequence identity and 60% sequence coverage. To compare SAUSA300_0905 with a genuine type IV adenylate cyclases, CyaB from *Y. pestis* was used as a query sequence to retrieve its closest homologs from the NCBI nr database. This yielded 562 sequences with a minimum of 30% sequence identity and 60% sequence coverage. But none of the CyaB homologs were from the Firmicutes group of bacteria. Next, multiple sequence alignments were performed within each group of proteins, and sequence logos were generated (Fig. 4*A*). A multiple sequence alignment was also performed across the two groups of proteins. This analysis revealed that the E*X*E*X*K signature motif of CYTH family protein (denoted by *gray stars* in Fig. 4*A*) is conserved in both groups of proteins. However, key residues required for the adenylate cyclase activity of type IV ACs, such as Lys-111 and Arg-113, which are essential for forming hydrogen bonds with the α- and β-phosphate groups of ATP, or Phe-5, Cys-83, Arg-113, and Glu-136 (*green stars* in Fig. 4*A*), which have been shown to be required for adenylate cyclase activity (8), are absent in SAUSA300_0905 and its homologs. On the other hand, a unique and highly conserved D*X*E*X*E*X*E motif (*yellow stars* in Fig. 4*A*) was identified within the C-terminal region of SAUSA300_0905 and its homologs that is absent from type IV ACs. Phyre2 was then used to generate a structure model of SAUSA300_0905 (using bh2851, a putative adenylate cyclase from *Bacillus halodurans*, with PDB code 2GFG as template, 94% of residues modeled at >90% confidence). This analysis suggested that the *S. aureus* protein SAUSA300_0905 does assume a fold typical for CYTH proteins with a tunnel-like structure similar to CyaB (PDB code 3N0Y) (Fig. 4*B*). Despite the similar structural fold, the absence of key residues required for adenylate cyclase activity and the presence of other conserved residues at the C terminus of the SAUSA300_0905 indicates that this protein is distinct from type IV adenylate cyclases and, therefore, may have a different enzymatic activity. Lastly, using the *E. coli* CyaA, *Bacillus anthracis* CyaA P40136, *Arthrospira platensis* CyaC O32393, *P. ruminicola* Cya O68902, and *R. etli* CyaC Q8KY20 proteins as representatives of type I, II, III, V, and VI ACs, respectively, in BLASTP searches, no proteins with significant similarity were found in Staphylococcaceae. These data suggest that no AC enzyme for the production of the classic signaling nucleotide 3′,5′-cAMP is encoded in *S. aureus*.

##### TrmDs from E. coli and M. tuberculosis Do Not Bind 3′,5′-cAMP

**FIGURE 5.**
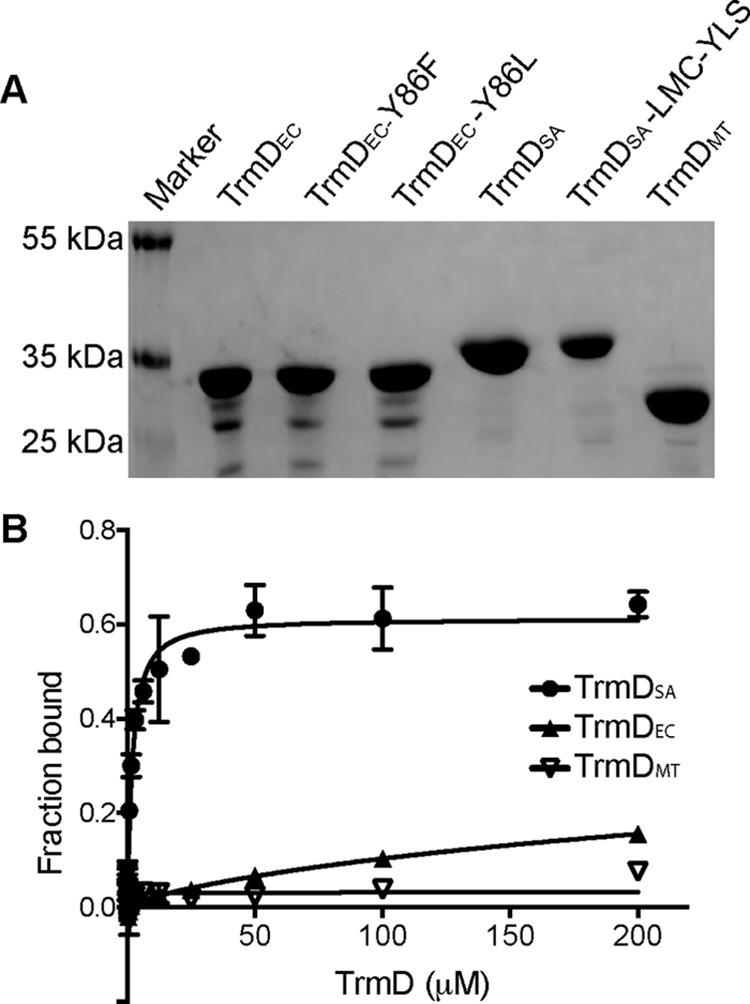
**The TrmD proteins from *E. coli* and *M. tuberculosis* did not bind 3′,5′-cAMP.** *A*, Coomassie-stained SDS-PAGE gel. About 7.5 μg of the indicated WT or mutant TrmD protein variants were separated on a 12% SDS-PAGE gel, and proteins were visualized by Coomassie staining. *B*, binding curve and *K_d_* determination between 3′,5′-cAMP and the *E. coli*, *M. tuberculosis*, and *S. aureus* TrmD proteins. Radiolabeled 3′,5′-cAMP and purified TrmD_SA_, TrmD_EC_, and TrmD_MT_ protein ranging from 1.5 nm to 200 μm were used in DRaCALAs, the average fraction-bound values and S.D. of four values were plotted, and the curve fitted, and *K_d_* value was determined as previously described (17).

Our results so far indicate that 3′,5′-cAMP can in *in vitro* assays competitively bind to the AdoMet substrate binding site of the *S. aureus* TrmD protein. On the other hand, we were unable to detect 3′,5′-cAMP in *S. aureus* extracts nor an enzyme that would be able to produce this signaling nucleotide. Hence, the interaction of 3′,5′-cAMP and the *S. aureus* TrmD protein may not be of physiological relevance. However, TrmD is a highly conserved protein and present in a large number of bacteria that have been experimentally shown to produce 3′,5′-cAMP. This raises the possibilities that in these organisms 3′,5′-cAMP could either be a competitive inhibitor of TrmD enzyme or alternatively that TrmD proteins evolved to discriminate between the 3′,5′-cAMP and AdoMet ligands. To address this question, TrmD proteins from the 3′,5′-cAMP producing γ-proteobacterium *E. coli* (TrmD_EC_) and the actinobacterium *M. tuberculosis* (TrmD_MT_) were chosen for further analysis. The genes coding for the corresponding TrmD proteins were cloned in the pET28b vector, and the proteins were expressed and purified as N-terminal His-tagged proteins (Fig. 5*A*). DRaCALAs were performed with radiolabeled 3′,5′-cAMP and the purified TrmD_EC_ and TrmD_MT_ proteins. This analysis revealed that neither protein could bind 3′,5′-cAMP as tightly as the *S. aureus* TrmD_SA_ protein, and due to this weak interaction no actual *K_d_* value could be determined using the DRaCALA method (Fig. 5*B*). These data suggest that TrmD proteins from organisms producing 3′,5′-cAMP are able to discriminate between 3′,5′-cAMP and the AdoMet ligand, likely preventing 3′,5′-cAMP to act as competitive inhibitor and blocking the essential functions of TrmD.

##### Tyr-86 Is Critical for Discriminating 3′,5′-cAMP from AdoMet in E. coli TrmD

**FIGURE 6.**
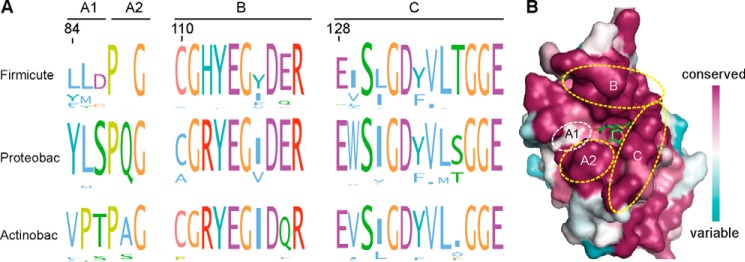
**Comparison of AdoMet binding site residues found in TrmD proteins from Firmicutes, γ-Proteobacteria, and Actinobacteria.** *A*, sequence logos motifs of AdoMet binding site residues found in TrmD proteins from Firmicutes, γ-Proteobacteria, and Actinobacteria. TrmD proteins from bacteria belonging to the Firmicutes, γ-Proteobacteria, and Actinobacteria were retrieved, and sequence alignments and logo motifs were prepared and displayed in the ClustalX default color scheme as described under “Experimental Procedures.” Amino acid residues forming the AdoMet binding site were identified and labeled as motifs A (further split into *A1* and *A2*), *B*, and *C*. Motifs *A2*, *B*, and *C* and are highly conserved between the three groups, whereas motif A1 is variable. Amino acid numbers indicated above the logo motif section is based on the *S. aureus* COL strain TrmD protein. *B*, ConSurf model of AdoMet binding site motifs of TrmD proteins. A multisequence alignment was generated for TrmD proteins found in Firmicutes, γ-Proteobacteria, and Actinobacteria and mapped using the ConSurf server onto the structure of the AdoMet-bound *H. influenza* TrmD protein (PDB code 1UAK). *Purple* represents high, *white* represents medium, and *turquoise* represents low conservation. The AdoMet ligand is shown as a stick model, and areas with motif A1, A2, B, and C amino acids are *circled*.

**FIGURE 7.**
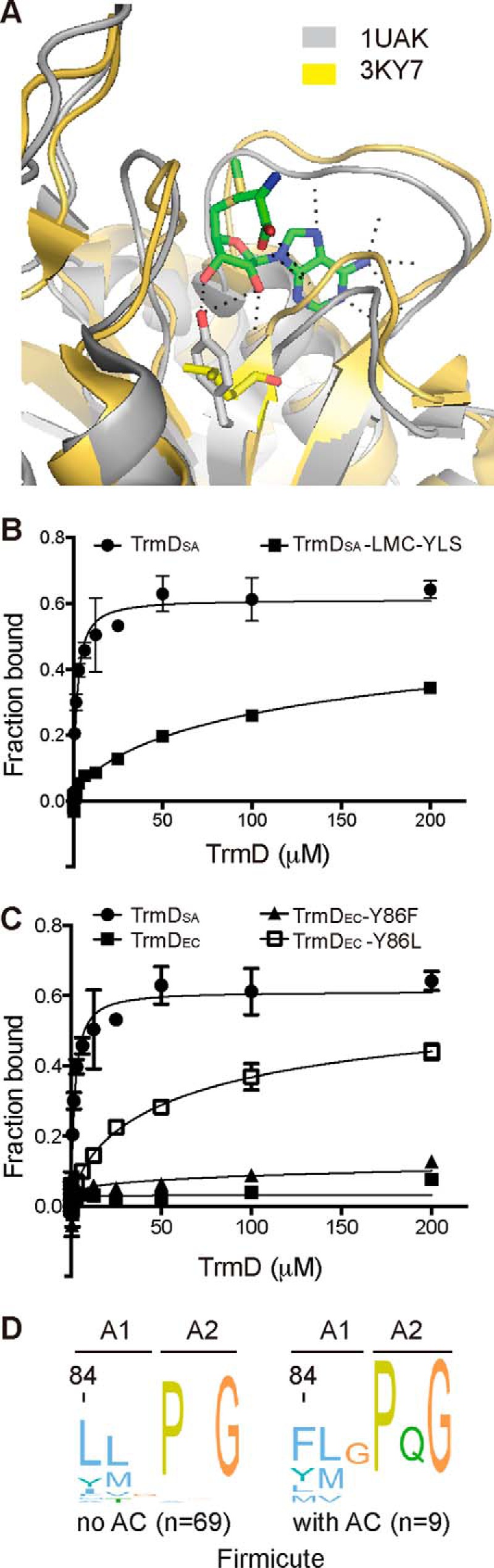
**Tyr-86 in *E. coli* TrmD is critical to discriminate between 3′,5′-cAMP and AdoMet.** *A*, comparison of the AdoMet-binding pockets of the *S. aureus* and *H. influenza* TrmD proteins. The apo structures of the *S. aureus* TrmD (PDB code 3KY7; shown in *yellow*) was overlaid in PyMOL with the AdoMet-bound structure of the *H. influenza* TrmD (PDB 1UAK, show in *gray*). A zoomed-in view of the ligand binding pocket is shown in schematic representation, with the AdoMet, Tyr-86 of TrmD_EC_, and Leu84 of TrmD_SA_ shown as a stick model. Tyr-86 of TrmD_EC_ formed two hydrogen bonds with 2′- and 3′-OH of the ribose ring of AdoMet, whereas Leu-84 of TrmD_SA_ probably cannot. *B* and *C*, binding curve and *K_d_* determination between 3′,5′-cAMP and WT *S. aureus* TrmD and the indicated variant (*B*) and WT *E. coli* TrmD protein and the indicated variants (*C*). Radiolabeled cAMP and purified TrmD_SA,_ or the TrmD_SA_-LMC-YLS variant TrmD_EC_ or the TrmD_EC_-Y86F and TrmD_EC_-Y86L variants ranging from 1.5 nm to 200 μm were used in DRaCALAs, the average fraction-bound values and S.D. of at least three values were plotted, the curve fitted, and *K_d_* value determined as previously described (17). *D*, Logo motifs of the TrmD protein A motif amino acids of representative Firmicutes. The TrmD protein sequences from 78 representative Firmicutes, as described in Galperin *et al.* (11), were retrieved and grouped into sequences from bacteria likely lacking a *bona fide* adenylate cyclase (no AC (*n* = 69)) or containing an adenylate cyclase (with AC (*n* = 9)). The sequences were aligned separately, and logo motifs were generated and displayed in the ClustalX default color scheme. The TrmD A motif was split as described in Fig. 6*A* into motifs A1 and A2, and amino acid numbers indicated *above each logo motif section* is based on the *S. aureus* strain COL TrmD protein sequence.

The difference in the capacity of the *S. aureus*, *E. coli*, and *M. tuberculosis* TrmD proteins to bind 3′,5′-cAMP indicates a critical difference in their primary sequences and tertiary structures. To gain further insight into this, each of the TrmD proteins was used to retrieve their close homologs from their respective groups (that is, Firmicutes, γ-Proteobacteria, and Actinobacteria). Multiple sequence alignments were performed individually within each group, and sequence logos were generated using Jalview (Fig. 6*A*) (28). This analysis revealed three highly conserved motifs termed here A, B, and C, which based on previous structural and functional analysis are known to form the active site and the AdoMet substrate binding pocket in TrmD proteins (Fig. 6*B*) (29, 30). Among these motifs, the motifs B and C with consensus sequences CG(H/R)YEG*X*D(E/Q)R and ExS*X*GD(Y/F)VL*X*GGE, respectively, are essentially the same in all three groups of bacteria (Fig. 6*A*). However, motif A differs significantly, with the consensus sequence YLSPQG in Proteobacteria, VPTPAG in Actinobacteria, and the degenerated consensus sequence (L/Y)(L/M)*X*P(Q/A)G in Firmicutes (Fig. 6*A*). In particular, the first three amino acids of motif A, referred to from here on out as motif A1, show very low conservation in the Firmicutes group of bacteria. To visualize the location of these motifs in a structural context, a multiple sequence alignment was created for the TrmD proteins from all three groups. The alignment was subsequently mapped on the crystal structure of the TrmD protein from *Hemophilus influenza* (PDB code 1UAK), a member of the γ-Proteobacteria group and displayed in the Consurf view (31). As expected, the three motifs, including the A1 motif, form the binding pocket for AdoMet on TrmD. The A1 motif area appears as white patch in the Consurf view, indicative of a lower conservation (Fig. 6*B*). To gain a better understanding of the location of the amino acids within the A1 motif in the context of the protein/AdoMet ligand interactions, a comparative structural alignment was performed with PyMOL using the available crystal structures of the *S. aureus* TrmD protein (PDB code 3KY7) and the *H. influenza* TrmD protein (PDB code 1UAK), the latter of which has been crystallized in the presence of AdoMet. As shown in the *H. influenza* TrmD-AdoMet complex structure, the Tyr residue at the beginning of the A1 motif (corresponding to Tyr-86 in *E. coli* TrmD_EC_) and highly conserved in Proteobacteria, forms hydrogen bonds with the 3′-OH and 2′-OH of the ribose ring in AdoMet (Fig. 7*A*). The corresponding 3′-OH group in 3′,5′-cAMP forms a phosphoester bond with the 5′-phosphate group (Fig. 7*A*) and hence would not be available for such a hydrogen bond interaction. This observation indicates that Tyr-86 in TrmD_EC_ might play a critical role in discriminating AdoMet from 3′,5′-cAMP by forming an additional hydrogen bond with AdoMet. Consistent with this idea, a Leu residue is present at the corresponding position in the A1 motif of the *S. aureus* TrmD_SA_ protein, which could not form a hydrogen bond with the 3′-OH of AdoMet, and this may allow TrmD_SA_ to bind AdoMet as well as 3′,5′-cAMP. To test this hypothesis, the LMC amino acid residues of the TrmD_SA_ A1 motif were replaced with YLS residues as found in TrmD_EC_. This variant was expressed and purified as N-terminal His-tagged protein (Fig. 5*A*). As assessed by DRaCALAs, this variant showed a decreased binding affinity for 3′,5′-cAMP (Fig. 7*B*). Conversely, the Tyr-86 residue in the TrmD_EC_ protein was replaced with a Phe (lacking the phenol hydroxyl group of Tyr) or Leu residue as present in TrmD_SA_, yielding the TrmD_EC_Y86F and TrmD_EC_Y86L variants, respectively (Fig. 5*A*). The TrmD_EC_Y86F variant was still unable to bind 3′,5′-cAMP (similar to WT TrmD_EC_); however, the TrmD_EC_Y86L variant had a much increased binding affinity for 3′,5′-cAMP (Fig. 7*C*). These findings were corroborated further by ITC experiments. Because of the weak binding, no *K_d_* values could be determined for the interaction between 3′,5′-cAMP and the WT TrmD_EC_ protein and the TrmD_EC_Y86F variant; however, the TrmD_EC_Y86L variant was able to bind 3′,5′-cAMP with a *K_d_* of 41.2 μm. On the other hand, this variant now had a decreased binding affinity for AdoMet with a *K_d_* of 69.9 μm, whereas the WT TrmD_EC_ and the TrmD_EC_Y86F variants had similar and high binding affinities for AdoMet with *K_d_* values of 21.5 μm and 27.9 μm, respectively. Taken together, these data indicate that the highly conserved Tyr-86 residue in *E. coli* TrmD and likely also in other γ-Proteobacteria is important for discriminating between 3′,5′-cAMP and AdoMet, preventing the binding of the former, which otherwise could competitively inhibit the enzyme.

##### Coevolution of TrmD with the Emergence of the 3′,5′-cAMP Signaling Pathway

The fact that TrmD proteins from bacteria that have been shown to produce 3′,5′-cAMP (*E. coli* and *M. tuberculosis*) do not bind 3′,5′-cAMP with high affinity, whereas TrmD from *S. aureus* tightly binds 3′,5′-cAMP raises the possibility that TrmD has evolved immunity to 3′,5′-cAMP in bacteria producing this signaling nucleotide. To test this hypothesis, the phylogeny of TrmD was analyzed and compared with that of adenylate cyclases. To do this we searched for homologs of *S. aureus* TrmD in the 555 complete bacterial and archaeal proteomes used by Galperin *et al.* (11) (supplemental Table S1). BLASTP queries identified a total of 503 homologs with a large taxonomic distribution. Specifically, homologues are found in one copy in all bacterial phyla (except for *Syntrophobacter fumaroxidans* MPOB that have two TrmD proteins: YP_847151 and YP_847109; supplemental Table S1). Close homologs are absent from Archaea, consistent with previous report that TrmD from Archaea is more similar to eukaryotic counterparts. A phylogenetic tree of TrmD homologs was built (supplemental Fig. S1) as well as all sequences were aligned, and careful inspection of the alignment showed that motifs B and C of the AdoMet binding site are well conserved among all TrmD homologs (supplemental Fig. S2). However, the first three residues of motif A are variable (supplemental Fig. S2). Based on the data of Galperin *et al.* (11) concerning the presence/absence of adenylate cyclase in bacterial proteomes, there appears to be a positive correlation between the presence or absence of ACs (and therefore the ability of bacteria to produce 3′,5′-cAMP) and the divergence of the first three residues of A motif in TrmD proteins, especially in γ-Proteobacteria and Firmicutes (Fig. 7 and supplemental Fig. S2). In fact, in the proteomes where an AC is present (such as in *E. coli*), the first residue of motif A is predominantly a tyrosine/phenylalanine (49.62% of the cases). However, in the proteomes where an AC is absent (such as in *S. aureus*) the first residue in motif A is in a large number of cases a leucine (46.08% of the cases). In particular, for Firmicutes bacteria, there appears to be a good correlation between the absence and presence of a tyrosine/phenylalanine residue in the first position of TrmD motif A1 and the absence and presence of an AC in an organism (Fig. 7*D*). Taken together, this indicates an underlying evolutionary pressure and protein evolution to prevent the binding of 3′,5′-cAMP to TrmD in organisms utilizing this cyclic-nucleotide as the signaling molecule.

## Discussion

Evidence whether or not the signaling nucleotide 3′,5′-cAMP is present in many of the well studied Firmicutes such as *S. aureus* has been elusive. In this study the *S. aureus* TrmD protein was identified as a 3′,5′-cAMP-binding protein, and 3′,5′-cAMP seems to bind competitively and with high affinity at the AdoMet substrate binding pocket (Fig. 1, *A* and *B*). However, using an LC-MS/MS analysis of bacterial extracts and based on other biochemical assays, we were unable to detect 3′,5′-cAMP or an active adenylate cyclase in *S. aureus* (Figs. 2 and 3). 3′,5′-cAMP is thus unlikely to be present in *S. aureus*. On the other hand, we found that TrmD proteins from the γ-proteobacterium *E. coli* and the actinobacterium *M. tuberculosis* do not bind 3′,5′-cAMP with high affinity. Subsequent bioinformatics, mutagenesis, and biochemical analyses demonstrated that residue Tyr-86 in *E. coli* TrmD plays a pivotal role in discriminating between 3′,5′-cAMP and the native substrate AdoMet. A further phylogenetic analysis suggests that amino acids in the substrate binding pocket of TrmD underwent an adaptive evolution to accommodate the presence of adenylate cyclases and the 3′,5′-cAMP signaling molecule.

Several lines of evidence argue for the absence of 3′,5′-cAMP in *S. aureus* and likely also in many other Firmicutes bacteria. The major function of 3′,5′-cAMP in bacteria, the carbon catabolite repression, is executed through a modified pathway in several of the model Firmicutes bacteria (*Bacillus*, *Listeria*, and *Staphylococcus*) (2). Although CRP-like transcription factors are present in Firmicutes, they often lack key residues known to be required for the binding of 3′,5′-cAMP (14). As shown in this study, ArcR, a CRP-family transcriptional factor in *S. aureus*, does not bind 3′,5′-cAMP as determined by DRaCALA (Fig. 1*C*). Using a very sensitive LC-MS/MS-based method, 3′,5′-cAMP could not be detected in *S. aureus* extracts prepared from cultures grown under several different conditions including micro-aerobic and catabolite repression conditions, where 3′,5′-cAMP was believed to be produced (Fig. 2) (12, 13). *In vitro* and *in vivo* experiments on the predicted adenylate cyclase SAUSA300_0905 (SACOL1008) enzyme indicated that this protein is not a genuine adenylate cyclase as this protein does not hydrolyze ATP nor produce 3′,5′-cAMP (Fig. 3, *A* and *B*). Close homologues to SAUSA300_0905 are found in many other bacteria belonging to the Firmicutes group. Despite the fact that this protein likely has a similar overall fold as type IV ACs, we identified in our bioinformatics analysis distinct sequence features in SAUSA300_0905 and its homologs compared with *bona fide* Class IV ACs (Fig. 4*A*). The physiological function of SAUSA300_0905, its enzymatic activity, and substrate specificity remain to be studied.

A genome wide DRaCALA screen for 3′,5′-cAMP-binding proteins using an *S. aureus* ORFeome library identified the essential tRNA methyltransferase TrmD_SA_ as 3′,5′-cAMP-binding protein (Fig. 1*A*). 3′,5′-cAMP binds with high affinity to TrmD_SA_ and competes for the binding with the native substrate AdoMet (Fig. 1*A*). The difference in binding affinity is indicated by the limited capacity of AdoMet to compete with 3′,5′-cAMP for binding to TrmD_SA_ (Fig. 1*B*). Given that TrmD binds its substrate tRNA species in an AdoMet-dependent manner (16), this difference in binding affinity suggests a potential inhibitory effect on the essential function of TrmD would 3′,5′-cAMP be present in *S. aureus*. As shown in this work, a tyrosine residue found at position 86 in the *E. coli* TrmD and highly conserved among TrmD proteins from γ-Proteobacteria, aids in the discrimination and preferential binding of AdoMet over 3′,5′-cAMP in *E. coli* (Figs. 6*A* and 7). This residue is absent in *S. aureus* and most other Firmicutes (Figs. 6*A* and 7*A*). However, some non-type Firmicutes strains, which are predicted to encode a genuine adenylate cyclase (11), such as *Clostridium acetobutylicum* ATCC 824, *Clostridium perfringens* str. 13, *Caldicellulosiruptor saccharolyticus* DSM 8903, *Desulfotomaculum reducens* MI-1, *Natranaerobius thermophilus* JW/NM-WN-LF, and their sub-strains, contain a Tyr or Phe residue at this position, suggesting a coupling of the presence of a functional adenylate cyclase and 3′,5′-cAMP production with the presence of a Tyr/Phe amino acid reside at this position (Fig. 7*D*). Altogether the findings presented in this study strongly suggest that 3′,5′-cAMP and a functional adenylate cyclase enzyme are absent in *S. aureus* and, although not all, likely also a large number of other bacteria belonging to the Firmicutes group.

**FIGURE 8.**
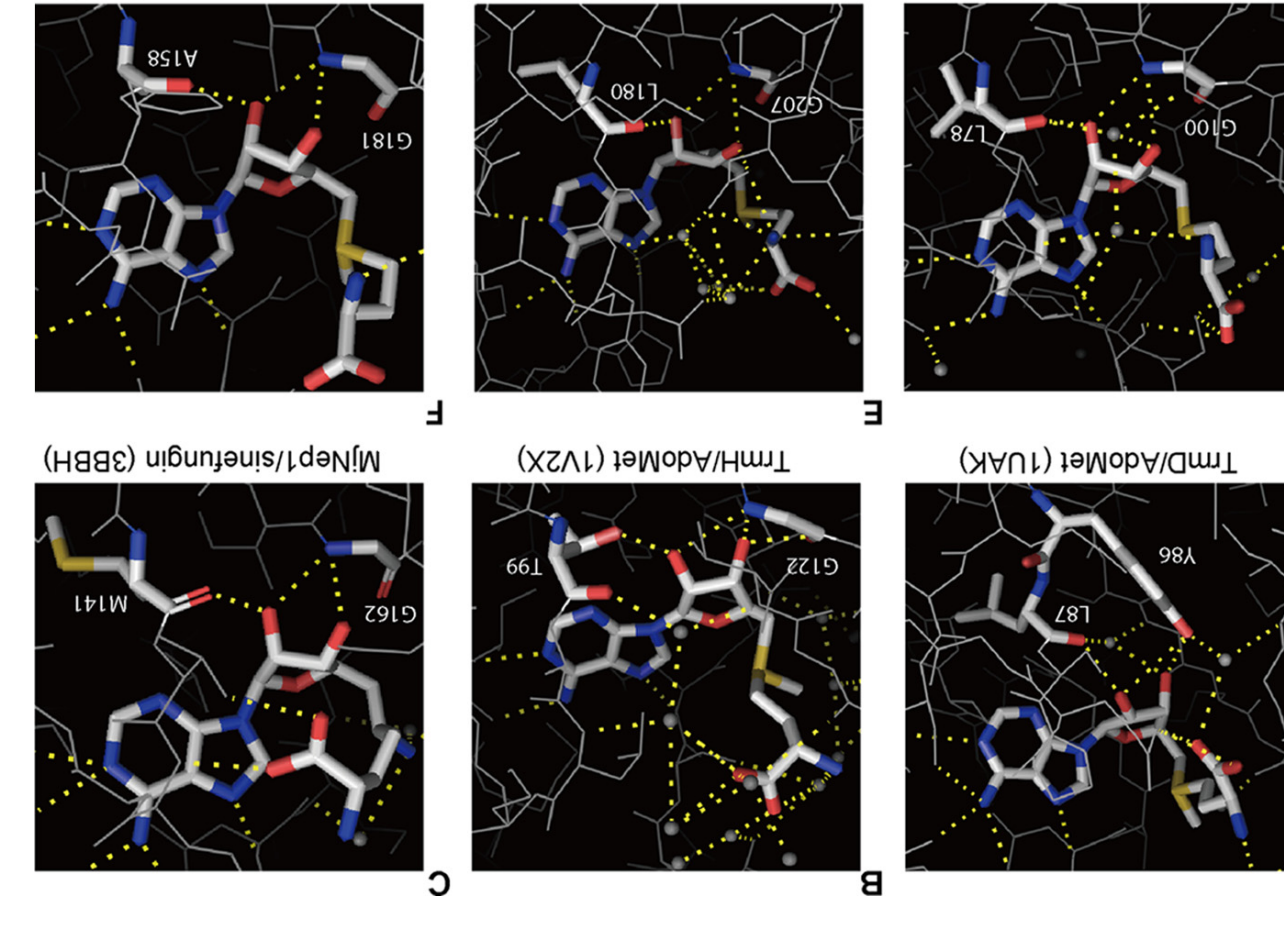
**Schematic representation of the hydrogen-bonding networks between *S*-adenosylmethionine or its analogs and bacterial tRNA/rRNA methyltransferases.** The substrate binding pockets are shown. *A*, tRNA MTases TrmD (tRNA (guanine-*N*(1)-)-methyltransferase, in complex with AdoMet, PDB code 1UAK), *B*, TrmH (tRNA (guanosine(18)-2′-*O*)-methyltransferase, in complex with AdoMet, PDB code 1V2X), *C*, MjNep1 (ribosomal RNA small subunit methyltransferase, in complex with sinefungin, PDB code 3BBH). *D*, TrmL (tRNA (cytidine(34)-2′-*O*)-methyltransferase in complex with AdoHcy, PDB code 1MXI). *E*, rRNA MTases ScNep1 (ribosomal RNA small subunit methyltransferase Nep1, in complex with AdoMet, PDB code 2V3K). *F*, RsmE (ribosomal RNA small subunit methyltransferase E, in complex with AdoMet, PDB code 2Z0Y). AdoMet, AdoHcy, and sinefungin and key protein amino acid residues involved in hydrogen bond (*yellow dotted lines*) formation with the 2′ and 3′-OH in the substrate are shown in stick models (carbon, *white*; oxygen, *red*; nitrogen, *blue*; sulfur, *orange*). *Small gray dots* indicate water molecules. Images were generated in PyMOL.

TrmD is a highly conserved tRNA methyltransferase found in all three domains of life that converts Gly-37 into m^1^G37 by transferring the methyl group from AdoMet to a subset of tRNA species (15, 32). This modification on tRNAs is essential for maintaining the correct reading frame during protein translation (15, 33). Abolishing the function of TrmD increases +1 frameshift events during protein translation, and growth defects have been observed in its absence in bacteria and yeast (15, 33–35). To bind the AdoMet substrate, TrmD proteins assumes a particular protein fold composed of a deep trefoil knot that is a characteristic of SPOUT family RNA methyltransferases (MTases). SPOUT family MTases methylate ribosomal RNAs or tRNAs on a base or ribose ring using AdoMet as the methyl donor (36). The conserved AdoMet binding site and unique protein fold of SPOUT MTases raises the question as to why other MTases from *S. aureus* were not identified as 3′,5′-cAMP-binding proteins. Several MTases have been crystallized in complex with AdoMet, its analogue sinefungin, or S-adonesyl-l-homocysteine (AdoHcy) (29, 37–39). Careful inspection of the substrate binding sites of these other MTases, namely the tRNA MTases TrmL (PDB code 1MXI), TrmH (PDB code 1V2X), MjNep1 (PDB code 3BBH), and the rRNA MTases ScNep1 (PDB code 2V3K) and RsmE (PDB code 2Z0Y) (Fig. 8), revealed that in these cases a main chain peptidyl amine of a highly conserved glycine residue forms hydrogen bonds with the 2′- and 3′-OH of AdoMet/AdoHcy/sinefungin. This difference might explain why only TrmD_SA_ was found to interact with 3′,5′-cAMP, as other MTases in *S. aureus* are likely able to discriminate between 3′,5′-cAMP and AdoMet.

In γ-Proteobacteria and Firmicutes, we found bioinformatically and experimentally a good correlation between the ability of TrmD proteins to discriminate between 3′,5′-cAMP and AdoMet and the presence of a tyrosine or phenylalanine residue at a position equivalent to position 86 in the *E. coli* TrmD protein (Figs. 6 and 7). These data suggest that TrmD proteins in bacteria producing 3′,5′-cAMP adapted to confer “immunity” to 3′,5′-cAMP, binding of which would otherwise inhibit the essential function of TrmD. However, this simple correlation was not obvious in all groups of bacteria. In particular, we found that TrmD proteins from the Actinobacteria group had a unique set of highly conserved amino acid residues VPT in the motif A1 (Fig. 6*A*). Considering the numerous adenylate cyclases reported in this group of bacteria (up to 16 ACs in *M. tuberculosis*) (1), it is possible that the TrmD proteins underwent a global whole-protein adaption and optimization to properly function in the unique physiological conditions imposed by these ACs. It remains to be investigated how these “unusual” TrmD proteins cope with the presence of physiological concentrations of 3′,5′-cAMP. Such studies could also have translational impact, as TrmD proteins from bacteria are distinct from those found in Eukaryotes and Archaea and thus remain attractive drug targets.

TrmD has been extensively studied, especially in pathogenic bacteria, where a number of biochemical and structural characterization were performed (16, 29) in order to pinpoint unique features that are drug-able. Indeed, the TrmD_MT_ protein was highlighted as an excellent drug target candidate in *M. tuberculosis* (40), and screens for small molecule TrmD enzyme inhibitor were performed (41, 42). Lahoud *et al.* (42) found that adenosine and methionine fragments of AdoMet preferentially inhibit bacterial TrmD proteins over those of eukaryotic-Archaea origins. Hill *et al.* (41) screened a large collection of compounds, and after further compound optimization, TrmD-specific inhibitor with nanomolar affinities were identified. In our study we surprisingly found that the second message 3′,5′-cAMP, a chemical analogue to AdoMet, specifically binds with a high affinity to TrmD from *S. aureus* and probably also several other Firmicutes groups of bacteria and could, therefore, also function as inhibitor in these bacteria.

In conclusion, this study provided several lines of evidence against the existence of 3′,5′-cAMP in *S. aureus* and, although not all, probably also many other Firmicutes. More importantly, TrmD_SA_ was found to bind 3′,5′-cAMP with high affinity, probably due to its chemical similarity to the native substrate AdoMet, whereas TrmD proteins from γ-Proteobacteria evolved to contain a Tyr/Phe residue at position 86, which confers immunity to a potentially detrimental effect of binding 3′,5′-cAMP. These findings provide a potential opportunity to develop drugs specifically targeting a number of pathogenic bacteria belonging to the Firmicutes, which would be especially important for notorious multidrug resistant superbugs such as Methicillin-resistant *S. aureus* strains.

## Experimental Procedures

### 

#### 

##### Bacterial Growth Conditions

*E. coli* strains were grown in Luria Bertani (LB). LB-M9 medium (49.3 mm Na_2_HPO_4_, 14.7 mm KH_2_PO_4_, 8.55 mm NaCl, 18.7 mm NH_4_Cl, 3.7 mm sodium succinate, 11.1 mm glucose, 2 mm MgSO_4_, 1% Tryptone, and 0.5% yeast extract) was used for the growth of the *E. coli S. aureus* ORFeome library strains. *S. aureus* strains were grown in TSB medium or B-medium (1% peptone, 0.5% yeast extract, 0.5% NaCl, 0.1% KH_2_PO_4_) supplemented with 25 mm glucose or sucrose (25). For micro-aerobic growth of *S. aureus*, cells were inoculated into 50-ml Falcon tubes filled up completely with TSB medium and incubated at 37 °C without agitation (43). Micro-aerobic growth was validated by adding 0.001% resazurin (Sigma) to the cultures as a redox indicator. When appropriate, cultures were supplemented with antibiotics indicated in Table 1.

**TABLE 1:** **Bacterial strains used in this study**

Strain	Relevant features	Reference
***Escherichia coli* strains**		
XL1 Blue	Cloning strain, TetR, ANG127	Stratagene
BL21(DE3)	*E. coli* strain used for protein expression, ANG191	Novagen
DHM1	*cyaA* mutant *E. coli* strain, ANG1310	(53)
ANG1824	XL1 Blue pET28b; KanR	Novagen
ANG1867	BL21 (DE3) pET28b; KanR	Novagen
ANG3168	BL21 (DE3) pET28b-His_6_-*cyaA*_EC_ (2–446); KanR	This study
ANG3412	DH5α pBAD33; CamR	(54)
ANG3413	XL1 Blue pBAD33-*cyaA*_EC_ (2–446)-His_6_; CamR	This study
ANG3414	XL1 Blue pBAD33-SAUSA300_0905-His_6_; CamR	This study
ANG3434	DHM1 pBAD33-*cyaA*_EC_ (2–446)-His_6_; CamR	This study
ANG3435	DHM1 pBAD33-SAUSA300_0905-His_6_; CamR	This study
ANG3436	DHM1 pBAD33; CamR	This study
ANG3290	T7IQ pVL791-His_6_-SACOL1008; CamR;CarbR	This study
ANG3650	BL21 (DE3) pET28b-His_6_-*trmD*_EC_; KanR	This study
ANG3651	BL21 (DE3) pET28b-His_6_-*trmD*_SA_; KanR	This study
ANG3682	XL1 Blue pET28b-His_6_-*trmD*_EC_-Y86F; KanR	This study
ANG3683	XL1 Blue pET28b-His_6_-*trmD*_EC_-Y86L; KanR	This study
ANG3684	XL1 Blue pET28b-His_6_-*trmD*_SA_-LMC-YLS; KanR	This study
ANG3685	XL1 Blue pET23b-His_6_-*trmD*_TB_; KanR	This study
ANG3686	BL21 (DE3) pET28b-His_6_-*trmD*_EC_-Y86F; KanR	This study
ANG3687	BL21 (DE3) pET28b-His_6_-*trmD*_EC_-Y86L; KanR	This study
ANG3688	BL21 (DE3) pET28b-His_6_-*trmD*_SA_-LMC-YLS; KanR	This study
ANG3689	BL21 (DE3) pET23b-His_6_-*trmD*_TB_; KanR	This study
ANG3895	T7IQ pVL791-His_6_-SACOL2653; CamR;CarbR	This study
ANG3999	XL1 Blue pET28b-His_6_-*cyaA*_EC_ (2–446); KanR	This study
ANG4001	XL1 Blue pET28b-His_6_-*trmD*_EC_; KanR	This study
ANG4002	XL1-Blue pET28b-His_6_-*trmD*_SA_; KanR	This study

***S. aureus* strains**		
JE2	USA300 MRSA strain (ANG2624)	(55)
NE1299	JE2 SAUSA300_0905::Tn; ErmR (ANG3894)	(55)

##### Strain and Plasmid Constructions

Bacterial strains and primers used in this study are listed in Tables 1 and 2, respectively. For construction of plasmids pET28b-His-*trmD*_EC_and pET28b-His-*trmD*_SA_ primer pairs, ANG1918/1919 and ANG1920/1921 were used to amplify the *trmD* genes using *E. coli* MG1655 or *S. aureus* LAC* chromosomal DNA as template. The PCR products were digested with NheI/EcoRI and ligated with plasmid pET28b that has been cut with the same enzymes. For construction of plasmids pET28b-His-*trmD*_EC_(Y86F) and pET28b-His-*trmD*_EC_(Y86L), primer pairs ANG1918/2152 and ANG1919/2153 primer pairs ANG1918/2154 and ANG1919/2155 were used in the first round of PCR. The resulting products were gel-purified and fused using primer pair ANG1918/1919. The final product was digested with NheI and EcoRI and ligated with plasmid pET28b that had been cut with the same enzymes. Plasmid pET28b-His-*trmD*_SA_(LMC-YLS) was constructed in a similar manner using primers ANG1920/2156 and ANG1921/2157 in the first PCR and primers ANG1920/1921 in the second PCR. The final product was digested with enzymes NheI and EcoRI and ligated with plasmid pET28b. For construction of plasmid pET23b-His-*trmD*_TB_, primer pair ANG2158/2159 and *M. tuberculosis* H37Rv chromosomal DNA were used to amplify the *trmD*_TB_ gene. The PCR product was digested with NdeI and HindIII and ligated with plasmid pET23b that had been cut with the same enzymes. For construction of plasmid pET28b-His_6_-*cyaA_EC_*(2–446) primer pair, ANG1788/1789 and *E. coli* MG1655 chromosomal DNA were used. The PCR product was digested with NheI and EcoRI and ligated with plasmid pET28b. For construction of plasmids pBAD33-*cyaA_EC_*(2–446)-His_6_ and pBAD33-SAUSA300_0905-His_6_, primer pairs ANG2003/2004 and ANG2005/2006 were used to amplify the *cyaA* or SAUSA300_0905 gene using *E. coli* MG1655 and *S. aureus* LAC* chromosomal DNA, respectively. The PCR products were digested with KpnI and XbaI and ligated with plasmid pBAD33 that has been cut with the same enzymes. All plasmids were initially recovered in *E. coli* strain XL-1 Blue, and sequences of inserts were confirmed by fluorescent automated sequencing (GATC Biotech). For protein expression and purification, the plasmids were introduced in *E. coli* strain BL21(DE3), yielding the strains as specified in Table 1.

**TABLE 2:** **Primers used in this study** Restriction sites are underlined.

Number	Name	Sequence
ANG1918	5-NheI-MG1655 TrmD	CTAGGCTAGCTGGATTGGCATAATTAGCCTGTTTCCTGAAATG
ANG1919	3-EcoRI-MG1655 TrmD	CGGAATTCttaCGCCATCCCATCATGTTTATGTTGCTG
ANG1920	5-NheI-SACOL1256 TrmD	CTAGGCTAGCAAAATTGATTATTTAACTTTATTTCCTG
ANG1921	3-EcoRI-SACOL1256 TrmD	CGGAATTCCTAACCTTTTTTCAATCCTATTTTATATCTTTC
ANG2152	EcTrmDY86F-R	TGACAGAAAAATCACCTTTGCGCCTTC
ANG2153	EcTrmDY86F-F	GTGATTTTTCTGTCACCACAGGGACGCAAG
ANG2154	EcTrmDY86L-R	TGACAGTAAAATCACCTTTGCGCCTTC
ANG2155	EcTrmDY86L-F	GTGATTTTACTGTCACCACAGGGACGCAAG
ANG2156	SaTrmDLMC-YLS-R	GGAGAGGTAAATAACGCGTGTTTGTTC
ANG2157	SaTrmDLMC-YLS-F	GTTATTTACCTCTCCCCACAAGGCGAGCCATTTTC
ANG2158	Rv2906c Forward	GCCATATGGCACACCACCACCACCACCACATGCGCATCGATATCGTGAC
ANG2159	Rv2906c Reverse	GCAAGCTTTCAGTCGGGGTGGGACAGGT
ANG2003	KpnI-CyaAEc.H6-F	GGGGTACCAGGAGATATACCATGTACCTCTATATTGAGACTC
ANG2004	XbaI-CyaAEc.H6-R	GCTCTAGAGGATCAGTGGTGGTGGTGGTGGTGCGAAAAATATTGCTGTAATAGC
ANG2005	KpnI-CyaZSa.H6-F	GGGGTACCAGGAGATATACCATGGCAACAAATCATGAAATAG
ANG2006	XbaI-CyaZSa.H6-R	GCTCTAGAGGATCAGTGGTGGTGGTGGTGGTGATTTATATTGTTTGAAAGTG
ANG1788	5-NheI-CyaA (2–446)	CTAGGCTAGCTACCTCTATATTGAGACTCTGAAACAGAGACTGG
ANG1789	3-EcoRI-CyaA (2–446)	CGGAATTCTCATTCCGAGAGATCGGGTGAAATCTGCGG

##### Protein Expression and Purification

*E. coli* BL21(DE3) strains (Table 1) were used for the expression and purification of His-CyaA_EC_(2–446), the His-tagged TrmD proteins, and its variants. One liter of cultures of the different strains were grown at 37 °C to an *A*_600_ of 0.5–0.7, protein expression was induced with 0.5 mm isopropyl 1-thio-β-d-galactopyranoside, and cultures were incubated overnight at 18 °C. For purification of His_6_-SACOL1008 and His_6_-SACOL2653, strain T7IQ containing plasmids pVL791-His_6_-SACOL1008 and pVL791-His_6_-SACOL2653 were grown at 30 °C overnight. The next day the cultures were diluted 1:50 into 1 liter of fresh LB medium and incubated for 4 h at 30 °C, and subsequently protein expression was induced with 1 mm isopropyl 1-thio-β-d-galactopyranoside for 4 h. Protein purifications by nickel-nitrilotriacetic acid affinity chromatography and size exclusion chromatography were performed as previously described (44). Protein containing fractions were pooled and concentrated to ∼10 mg/ml using 10-kDa cut-off centrifugal filters. Protein concentrations were determined using the BCA protein assay kit from Pierce. The purity of the purified proteins was assessed on Coomassie-stained gels after separation of the indicated amounts of protein on 12% SDS-PAGE gels.

##### Synthesis of ^32^P 3′,5′-cAMP

^32^P-Labeled 3′,5′-cAMP was synthesized from [α-^32^P]ATP (PerkinElmer Life Sciences) by incubating 55.5 nm [α-^32^P]ATP with 20 μm purified His-CyaA_EC_(2–446) protein in 40 mm Tris, pH 7.5, 100 mm NaCl, 10 mm MgCl_2_ buffer overnight at 37 °C. The sample was subsequently incubated for 10 min at 95 °C, the denatured His-CyaA_EC_(2–446) protein was removed by centrifugation at 17,000 × *g* for 5 min, and the supernatant was transferred to a new tube and stored at −20 °C. The conversion of [α-^32^P]ATP to [α-^32^P]cAMP was estimated to be at least 97%, as assessed by TLC using H_2_O/ethanol/ammonium bicarbonate (30%:70%:0.2 m) as the mobile phase and densitometry analysis, which was performed as described previously (18).

##### Differential Radial Capillary Action of Ligand Assay and Screen for 3′,5′-cAMP-binding Proteins

An *S. aureus* ORFeome library allowing the expression of 2337 N-terminally His-MBP-tagged *S. aureus* strain COL proteins in *E. coli* was utilized to identify potential 3′,5′-cAMP target proteins. The construction and use of this ORFeome library has been described previously (19, 20). Protein expression and the preparation of whole cell lysates and the subsequent DRaCALA were performed as previously described, with the modification that ^32^P-labeled 3′,5′-cAMP was used as the nucleotide ligand (17–19). For the determination of *K_d_* values by DRaCALA, 2-fold serial dilutions of purified His-TrmD_SA_, His-TrmD_EC_, His-TrmD_TB_, or the different variants were prepared in binding buffer (40 mm Tris, pH 7.5, 100 mm NaCl, 10 mm MgCl_2_) starting at a concentration of 200 μm and subsequently mixed with ∼2 nm ^32^P-labeled 3′,5′-cAMP. The mixtures were incubated for 5 min at room temperature before spotting 2.5 μl of the reactions on nitrocellulose membranes (Amersham Biosciences Hybond-ECL; GE Healthcare). The fraction of ligand bound and *K_d_* values were calculated as previously described (17). For nucleotide competition assays, the specified purified protein at a final concentration of 100 μm was incubated with ∼2 nm ^32^P-labeled 3′,5′-cAMP in the presence of 400 μm concentrations of the competitor nucleotides AdoMet, 3′,5′-cAMP, 3′,5′-cGMP, c-di-AMP, c-di-GMP, ATP, ADP, AMP, or 2′,3′-cAMP. The reactions were incubated for 5 min at room temperature, 2.5 μl was spotted onto nitrocellulose membranes, and fraction-bound values were determined as described above.

##### In Vitro Adenylate Cyclase Assay

The purified His_6_-SACOL1008 protein was tested for potential adenylate cyclase activity using previously described *in vitro* assay systems (8, 26). The purified His-CyaA_EC_(2–446) protein was used as the positive control, and enzyme assays were set up in three different buffer systems. Buffer 1 consisted of 40 mm Tris, pH 7.5, 100 mm NaCl, and 10 mm MgCl_2_ and was previously used to measure the activity of the *E. coli* CyaA enzyme (26). Buffer 2 consisted of 50 mm Tris, pH 8.8, 20 mm MgCl_2_, and 1 mm DTT, and buffer 3 was similar to buffer 2 but 20 mm MnCl_2_ was replaced with 20 mm MgCl_2_. Buffers 2 and 3 have been previously used to assess the activity of type IV ACs (8). 4 μm purified protein was used in a 10-μl reaction volume with 333.3 nm [α-^32^P]ATP added. The reactions were incubated at 37 °C for 1 h or overnight, heat-inactivated, and subsequently analyzed by TLC as described above.

##### In Vivo Adenylate Cyclase Activity Assay

The ability of SAUSA300_0905 (SACOL1008 homolog) to synthesize 3′,5′-cAMP in *E. coli* was tested by introducing the plasmid pBAD33-SAUSA300_0905-His_6_ into the *E. coli cyaA* mutant strain DHM1. Plasmids pBAD33-*cyaA*_EC_(2–446)-His_6_ and pBAD33 were also introduced into strain DHM1, and the resulting strains were used as positive and negative controls, respectively. After transformation into DHM1, ∼30 colonies from each transformation plate were inoculated into LB medium and incubated at 37 °C overnight. The next morning they were subcultured in fresh LB medium at 37 °C and grown to an *A*_600_ of ∼0.5. One ml of each culture was harvested and adjusted to *A*_600_ of 5, and 5 μl were spotted on LB plates supplemented with 0.02% arabinose and 40 μg/ml X-Gal and the appropriate antibiotics. Plates were incubated overnight at 37 °C, and photos were taken with a Nikon camera.

##### Preparation of Cell Extract and Detection of Nucleotides by LC-MS/MS

*S. aureus* strains JE2 and JE2 Tn::SAUSA300_0905 (NE1299, ANG3894) were grown overnight in TSB medium as well as in B-medium supplemented with 25 mm concentrations of either glucose or sucrose (for stationary phase samples). The next day cultures were also back-diluted 1:50 into fresh medium and incubated for 3 h at 37 °C (for exponential phase samples). Bacterial cells corresponding to a 1-ml culture of *A*_600_ of 10 were harvested by centrifugation, and cell extracts for LC-MS/MS analysis were prepared as described below. For cells grown under micro-aerobic conditions, overnight cultures were diluted 1:50 into 50-ml Falcon tubes filled up to the top with fresh TSB medium and incubated at 37 °C without agitation for 24 or 48 h (for exponential and post-exponential phase samples). To ensure that the bacteria did not aerobically respire under these growth conditions, resazurin was added to the cultures at a final concentration of 0.001% w/v as previously described (43). The reduction potential of resazurin at pH 7.0 and 25 °C is +380 mV, sitting between oxygen gas (+820 mV) and cytochromes (+290 to +80). This makes this compound suitable for detecting aerobic respiration activity or lack thereof, as the former would lead to the reduction of resazurin to resorufin and a blue to pink color change (45). Next, bacteria were collected by centrifugation, and the pellets were suspended immediately in 1 ml of nucleotide extraction buffer containing acetonitrile-methanol-water (2:2:1, v/v) and heated for 15 min at 95 °C to minimize the effect of oxygen on the cell physiology and metabolites. Six samples were prepared for each culture condition for strains JE2 and JE2 Tn::SAUSA300_0905. For three of these samples the extraction buffer was spiced with 92.8 ng/ml isotope-labeled 3′,5′-cAMP. To generate the isotope-labeled 3′,5′-cAMP, 5 mm ^13^C,^15^N-ATP was converted into ^13^C,^15^N-cAMP with 5 μm *E. coli* CayA(2–446) in 40 mm Tris. pH 7.5, 100 mm NaCl, and 10 mm MgCl_2_ buffer, and the sample was incubated at 37 °C overnight. The conversion rate was determined as 93.4% by LC-MS/MS analysis. *S. aureus* extracts were prepared, and 3′,5′-cAMP and 2′,3′-cAMP was detected by LC-MS/MS as described previously (24).

##### K_d_ Determination by ITC

A MicroCal iTC200 instrument (GE Healthcare) was used to determine the disassociation constants of 3′,5′-cAMP or AdoMet and the *E. coli* or *S. aureus* wild-type TrmD proteins or TrmD variants. To minimize the dilution effect, the purified TrmD proteins were dialyzed for 24 h at 4 °C against 4 liters of binding buffer (40 mm Tris, pH 7.5, 100 mm NaCl, 10 mm MgCl_2_, 5% v/v glycerol). Subsequently, the samples were spun down at 17,000 × *g* at 4 °C for 10 min to remove any insoluble material, the supernatant was transferred to new tubes, and the protein concentrations were measured using a BCA assay kit (Pierce). An aliquot of the dialysis buffer was used to make 1 mm 3′,5′-cAMP and AdoMet solutions and also used to set the TrmD proteins to a concentration of 100 μm. After initial trials, the MicroCal iTC200 was set to a reference power of 6 μcal/s, a stirring speed of 500 rpm, and a temperature of 25 °C, and 20 injections were made at 180-s intervals. At least two technical replicates were performed with each TrmD protein. As the negative control, the ligands were titrated against the dialysis buffer, and the obtained values were subtracted from the experimental data. Curve-fitting, data analysis, and *K_d_* calculations were performed using the Origin program.

##### Western Blot

The expression of SAUSA300_0905-His_6_ and CyaA_EC_(2–446)-His_6_ from the pBAD33 vectors in DHM1 was confirmed by Western blot. Briefly, cultures of strain DHM1 containing the different pBAD33-derived vectors were grown overnight in LB medium at 30 °C. The next day the overnight cultures were induced with 0.02% arabinose for 3 h. Bacteria from 1-ml culture aliquots before and after the induction were collected by centrifugation, and cells were suspended in 1× SDS sample buffer to get a final *A*_600 nm_ of 40. Samples were heated for 10 min at 95 °C, and proteins separated on a 12% SDS-PAGE gel. Proteins were then transferred to a PVDF membrane, and His-tagged proteins were detected using a monoclonal anti-poly-His-peroxidase antibody (Sigma A-7058).

##### Sequence and Structure Analysis

Homologs of the *S. aureus* protein SAUSA300_0905, the *Y. pestis* CyaB protein, and the TrmD proteins from *E. coli*, *S. aureus*, and *M. tuberculosis* proteins were identified as follows. For SAUSA300_0905 and CyaB from *Y. pestis*, the respective protein sequences were used as query sequences in BLASTP searches in the NCBI non-redundant (nr) protein sequence database using default settings. For the TrmD proteins, the respective protein sequences were used as query sequence in a BLASTP search confined to their respective groups of bacteria, namely *E. coli* TrmD for γ-Proteobacteria, *M. tuberculosis* TrmD for Actinobacteria, and *S. aureus* TrmD for Firmicutes. Subsequently, the identified protein homologs with a maximum expect (e) values below 3e-04 and a minimum sequence coverage and sequence identity of 60 and 30%, respectively, were retrieved and used for further analysis in Jalview (28). A multisequence alignment was generated for each group of proteins by running 20 iterations, and a conserved logo-sequence was generated with Cluster Omega (46). To compare the sequences of the SAUSA300_0905 and CyaB homologs or the *E. coli*, *S. aureus*, and *M. tuberculosis* TrmD homologs, the respective groups of sequences were combined, and a multi-sequence alignment and/or a logo-sequence was generated as described above. A multiple sequence alignment of all TrmD proteins was also generated and subsequently used as the input sequence on the ConSurf server to visualize the AdoMet binding site in a structural context (31). To this end, chain A of the AdoMet-bound *H. influenza* TrmD protein was used as the structural template (PDB code 1UAK). PyMOL (v1.7.4.4 Edu Enhanced for Mac OS X, Schrödinger, LLC.) was used to display the ConSurf data and also for the structural comparison of the AdoMet binding site of the *S. aureus* TrmD (PDB code 3KY7) and *H. influenza* TrmD (PDB code 1UAK) proteins. A structural model of SAUSA300_0905 was generated in Phyre2 (47) and viewed in PyMOL.

##### Phylogeny Analysis of TrmD and Adenylate Cyclases

A local protein database containing the 555 complete bacterial and archaeal proteomes used by Galperin *et al.* (11) in his study on the distribution of bacterial signal transduction systems was built. This database was queried with the BLASTP program (default parameters; Ref. 48) using the full-length sequence of TrmD protein of *S. aureus* strain N315 as a seed (Ref_seq: NP_374356, Locus_tag: SA1083). The distinction between homologous and non-homologous sequences was assessed by visual inspection of the BLASTP output (no arbitrary cut-offs on E-value or score). To ensure the exhaustive sampling of homologs, iterative BLASTP queries were performed using homologs identified at each step as new seeds. The absence of a homolog in any complete proteome in the local database was systematically verified by TBLASTN queries against the nucleotide sequence of the corresponding genome. For each candidate protein, the retrieved homologs were added to the dataset. The retrieved sequences were aligned using MAFFT v7.045b (default parameters; Ref. 49). Regions where the homology between amino acid positions was doubtful were removed using the BMGE software (BLOSUM30 option; Ref. 50). Bayesian analyses were performed using MrBayes version 3.2.2 (51) with a mixed model of amino acid substitution including a gamma distribution (4 discrete categories) and an estimated proportion of invariant sites. MrBayes was run with 4 chains for 1 million generations, and trees were sampled every 100 generations. To construct the consensus tree, the first 2000 trees were discarded as “burn in” (51). The Sequence-logos of TrmD the alignments were generated using Phylo-mLogo visualization tool to highlight the three motifs involved in the AdoMet binding (52).

## Author Contributions

Y. Z. and A. G. designed the study, acquired funding, and wrote the manuscript. Y. Z. and L. E. B. acquired the experimental data, Y. Z. and R. A. performed the bioinformatics analyses, Y. Z., R. A., L. E. B., J. C., V. K., and A. G. analyzed the data.

## Supplementary Material

Supplemental Data

## Footnotes

3 The abbreviations used are:

CCR: carbon catabolite repression
CRP: catabolite receptor protein
AC: adenylate cyclases
AdoMet: *S*-adenosyl-l-methionine
DRaCALA: differential radial capillary action of ligand assay
ITC: isothermal titration calorimetry
TSB: tryptic soy broth
MTase: methyltransferase
AdoHcy: adonesyl-l-homocysteine.
