# Supplementary material for: Evolutionary Adaptation of the Essential tRNA Methyltransferase TrmD to the Signaling Molecule 3′,5′-cAMP in Bacteria
Source: J Biol Chem. 2016 Nov 23;292(1):313–27. doi: 10.1074/jbc.M116.758896 (PMC5217690; doi:10.1074/jbc.M116.758896)
Supplement: Supplemental Data [file supp_292_1_313__index.html]

Evolutionary Adaptation of the Essential tRNA Methyltransferase TrmD to the Signaling Molecule 3,5-cAMP in Bacteria — Evolutionary Adaptation of the Essential tRNA Methyltransferase TrmD to the Signaling Molecule 3′,5′-cAMP in Bacteria — Protein Adaption with the Emergence of 3′,5′-cAMP — Supplemental Data 

# Evolutionary Adaptation of the Essential tRNA Methyltransferase TrmD to the Signaling Molecule 3′,5′-cAMP in Bacteria

## Supplemental Data

- Figure S1 (.pdf, 290 KB) - Phylogenetic tree of TrmD homologs.
- Figure S2 (.pdf, 11.3 MB) - Multiple sequence alignment of TrmD protein and its homologs.
- Table S1 (.xlsx, 318 KB) - List of TrmD homologs found in 555 complete proteomes.
- Supplemental Material (.docx, 96 KB) - Figure and Table legends
