# Supplementary figures and images for: Evolutionary Adaptation of the Essential tRNA Methyltransferase TrmD to the Signaling Molecule 3′,5′-cAMP in Bacteria
Source: J Biol Chem. 2016 Nov 23;292(1):313–27. doi: 10.1074/jbc.M116.758896 (PMC5217690; doi:10.1074/jbc.M116.758896)

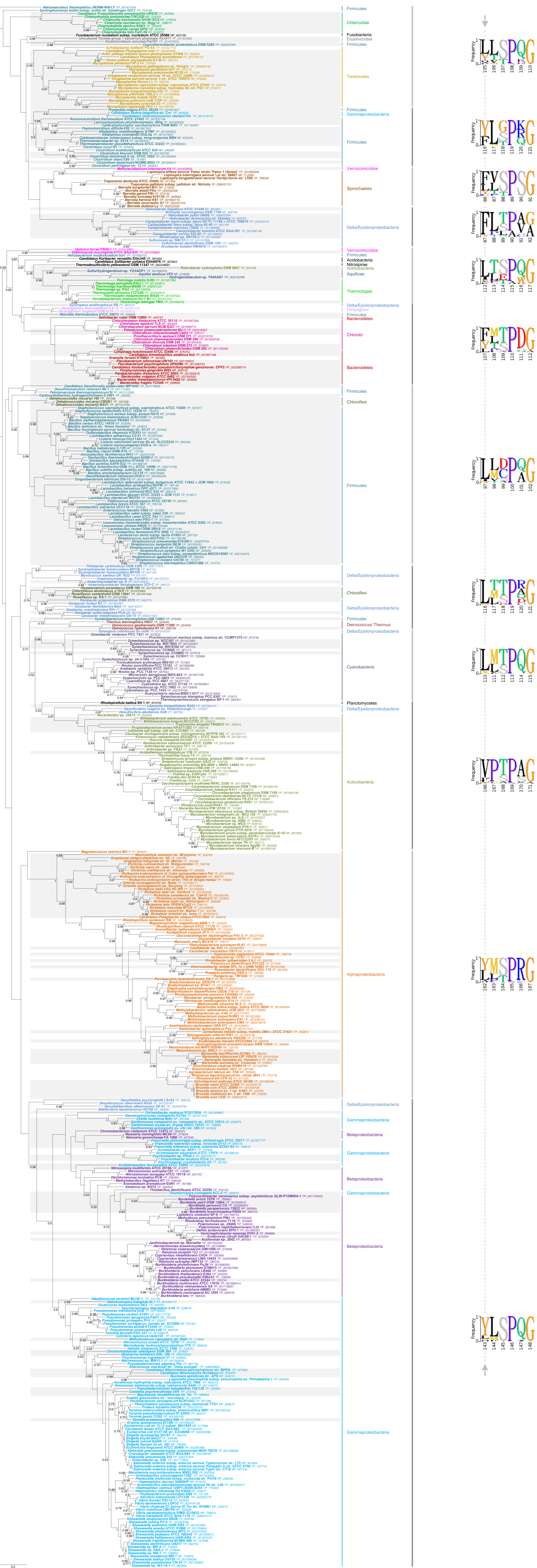

Supplement: Supplemental Data [file 10.1074_M116.758896_jbc.M116.758896-1.pdf]

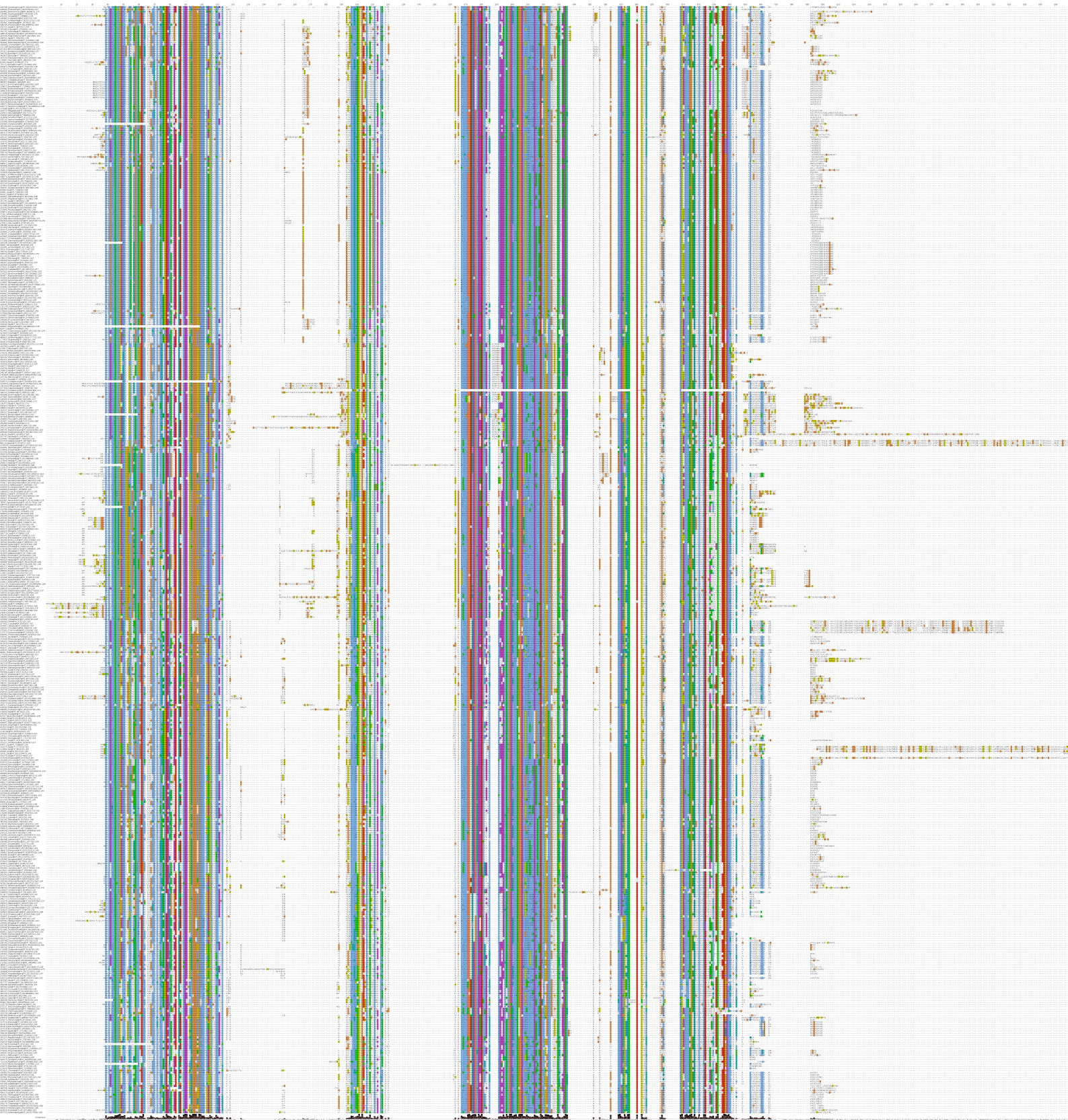

Supplement: Supplemental Data [file 10.1074_M116.758896_jbc.M116.758896-2.pdf]
