# Supplementary material for: Evolutionary Adaptation of the Essential tRNA Methyltransferase TrmD to the Signaling Molecule 3′,5′-cAMP in Bacteria
Source: J Biol Chem. 2016 Nov 23;292(1):313–27. doi: 10.1074/jbc.M116.758896 (PMC5217690; doi:10.1074/jbc.M116.758896)
Supplement: Supplemental Data [file 10.1074_M116.758896_jbc.M116.758896-4.docx]

**Supplemental material**

**Table S1.** List of TrmD homologs found in 555 complete proteomes. For each entry the ref_seq database accession number, the motif involved in AdoMet and/or cAMP binding and the presence/absence of adenylate cyclases according to the analysis of Galperin *et al.* (1) are provided. Genomes that lack genes encoding for adenylate cyclase (AC) are highlighted in grey. Numbers with asterisks indicate the presence of proteins with highly divergent and/or truncated domains, and not included in the total count but listed at the end the list.

**Figure S1.** Phylogenetic tree of TrmD homologs. Unrooted Bayesian phylogenetic tree of TrmD homologs (503 sequences, 185 positions). Number at nodes indicates posterior probabilities (PP) computed by Mrbayes. Only posterior probabilities values greater than 0.5 are shown. The scale bars represent the number of substitutions per site. Sequence-logo of the motif A involved on AdoMet biding is represented in the right panel using Phylo-mLogo. The logos are generated based on the monophyletic group in the tree. Genomes that lack genes encoding for adenylate cyclase (AC) are highlighted in grey. The dotted line in grey indicates the Tyr86 residue (position in *E. coli*).

**Figure S2.** Multiple sequence alignment of TrmD protein and its homologs. The sequences of TrmD homologs identified from 555 complete proteomes were aligned using MAFFT v7.045b (2) and edited with Jalview (3).

1. Galperin, M. Y., Higdon, R., and Kolker, E. (2010) Interplay of heritage and habitat in the distribution of bacterial signal transduction systems. *Mol. Biosyst.* **6**, 721-728

2. Katoh, K., and Standley, D. M. (2013) MAFFT multiple sequence alignment software version 7: improvements in performance and usability. *Mol. Biol. Evol.* **30**, 772-780

3. Waterhouse, A. M., Procter, J. B., Martin, D. M., Clamp, M., and Barton, G. J. (2009) Jalview Version 2--a multiple sequence alignment editor and analysis workbench. *Bioinformatics* **25**, 1189-1191
